# Assessment of Real-World Use of Behavioral Health Mobile Applications by a Novel Stickiness Metric

**DOI:** 10.1001/jamanetworkopen.2020.11978

**Published:** 2020-08-03

**Authors:** Andrew D. Carlo, Reza Hosseini Ghomi, Brenna N. Renn, Michael A. Strong, Patricia A. Areán

**Affiliations:** 1 Department of Psychiatry and Behavioral Sciences, University of Washington School of Medicine, Seattle; 2 Department of Neurology, University of Washington School of Medicine, Seattle; 3 Perelman School of Medicine, Department of Psychiatry, University of Pennsylvania, Philadelphia

## Abstract

This cross-sectional study uses a novel “stickiness” measure to evaluate which mobile applications for smoking cessation, meditation, and other behavioral health areas are associated with long-term engagement.

## Introduction

Digital health treatments for individuals with behavioral health problems are increasing rapidly in number.^1,2^ Studies to date have demonstrated that longitudinal patient engagement is challenging,^3,4^ making it unlikely for most applications (hereafter, *apps*) to effect real-world change. In this cross-sectional study, we describe usage patterns of popular mobile behavioral health apps and identify characteristics of those that are most continually accessed (also known as the *stickiest*),^5^ with the aim of informing future research on behavioral health app engagement.

## Methods

This cross-sectional study was granted exemption from review and informed consent by the University of Washington institutional review board as it did not involve human participants. The study follows the Strengthening the Reporting of Observational Studies in Epidemiology (STROBE) reporting guideline for cross-sectional studies.

This study builds on a previously published study^1^ that curated a sample of apps listed on 5 online rating frameworks, including 201 apps from iOS (Apple) and 152 apps from Google Play (Alphabet), excluding those (1) not designed to treat a behavioral health disorder or (2) not promoting a specific behavioral health treatment or technique. Download and utilization data for all apps were procured between September 21 and October 21, 2018, from Priori Data, a mobile app market research firm.^1^ For this analysis, we applied additional selection criteria excluding apps with (1) availability restricted to 1 of the 2 leading marketplaces (either Apple iOS or Google Play), (2) fewer than 10 000 total global downloads since first tracked on Priori Data, or (3) fewer than 1000 total global monthly active users over the 30 days preceding data acquisition.

We specify a novel stickiness metric derived from the quotient of 2 of our app parameters: monthly active users and total downloads. This metric, termed *number of monthly active users per normalized total downloads*, estimates the number of active app users per normalized download, with a higher number indicating greater stickiness. Of note, total download figures were normalized at the month level to account for variation in the lengths of time that apps have been available on the marketplaces. Additionally, the total download and stickiness metric values for each app across both marketplaces were summed to create composite values. Calculations were conducted using Excel version 16.36 (Microsoft). Data were analyzed from November 2019 to May 2020.

## Results

A total of 46 apps met inclusion criteria for this analysis. Total downloads and stickiness metric values for the 10 most downloaded apps^1^ are shown in Table 1. All of the 10 most downloaded apps were from private developers; 6 were for meditation (Headspace; Calm; Relax Meditation P; Insight Timer; Stop, Breathe & Think; and Sanvello for Stress & Anxiety), 2 focused on cognitive training (Peak and Lumosity), and none were developed for specific behavioral health conditions. Among the 46 included apps, the median (range) stickiness score was 1.76 (0.26-9.87) and the mean (SD) stickiness score was 2.23 (1.84). Only 3 of the 10 most downloaded apps had stickiness metric scores above the mean (Lumosity: stickiness score, 2.63; Headspace: stickiness score, 3.18; Relax Meditation P: stickiness score, 2.48). Table 2 shows the total downloads and stickiness metric values for the 10 stickiest apps, only 2 of which were ranked in the top half with regard to total downloads (Smiling Mind: 2.2 million total downloads; stickiness score, 3.44; Headspace: 26 million total downloads; stickiness score, 3.18). Among the top 10 stickiest apps, 9 were from private developers (CogniFit, Muse, Stop Smoking in 2 Hours, Daybreak, Youper, PanicShield, Smiling Mind, Relax and Sleep Well, and Headspace), 3 targeted alcohol or cigarette use (My QuitBuddy, Stop Smoking in 2 Hours, and Daybreak), 2 included cognitive behavioral therapy skills (Youper and PanicShield), and 5 focused on meditation (Muse, Youper, Smiling Mind, Relax and Sleep Well Hypnosis, and Headspace).

**Table 1.** The 10 Most Downloaded Mobile Behavioral Health Applications on the Apple App Store and Google Play Marketplaces

Name	Developer	Type	Downloads		Stickiness	
			Total No., millions	Rank^a^	Score^b^	Rank^a^
Peak - Brain Training	brainbow	Cognitive training	42	1	2.13	17
Lumosity: Brain Training^c^	Lumos Labs	Cognitive training	27	2	2.63	13
Headspace^c^	Headspace	Meditation	26	3	3.18	10
Calm	Calm.com	Meditation	25	4	2.11	18
Relax Meditation P: Mindfulness Sounds White Noise^c^	Ipnos Software	Meditation	15	5	2.48	14
Fabulous - Daily Self Care	Fabulous	Health coach, behavior tracking	6	6	0.57	45
Daylio Journal	Relaxio s.r.o.	Mood tracking	6	7	0.90	37
Insight Timer - Meditation App	Insight Network	Meditation	5	8	2.19	16
Stop, Breathe & Think	Stop, Breathe & Think	Meditation	4	9	1.82	21
Sanvello for Stress & Anxiety	Sanvello Health	Meditation, mood tracking, peer support	3	10	1.03	35

^a^ A total of 46 apps with more than 1000 monthly active users and 10 000 total downloads on both marketplaces were included.

^b^ The stickiness score is the sum of the stickiness subscores for each app on the marketplaces. The stickiness subscores are the quotient of monthly active users and normalized total downloads. Normalized total download figures are the quotient of total downloads and the number of months since an app first appeared on the marketplaces (it is often the case that apps are not released on both marketplaces simultaneously). Higher stickiness scores indicate stickier apps.

^c^ Indicates an app with a stickiness score above the mean (SD) score for all included apps (2.23 [1.84]).

**Table 2.** The 10 Stickiest Mobile Behavioral Health Applications on the Apple App Store and Google Play Marketplaces

Name	Developer	Type	Downloads		Stickiness	
			Total No., millions	Rank^a^	Score^b^	Rank^a^
CogniFit - Brain Training	CogniFit	Cognitive training	207 000	34	9.87	1
Muse: Meditation & Sleep	InteraXon	Meditation	174 000	37	6.43	2
My QuitBuddy	Australian National Preventive Health Agency	Smoking cessation	446 000	29	6.22	3
Stop Smoking in 2 Hours	Juice Master	Smoking cessation	103 000	41	5.40	4
Daybreak - Alcohol Support	Hello Sunday Morning	Modify alcohol use	34 000	46	4.11	5
Youper	Youper	Mood tracking, meditation, CBT, ACT	266 000	33	3.93	6
PanicShield - Panic Attack Aid	Eddie Liu	Deep breathing, CBT	35 000	45	3.75	7
Smiling Mind^c^	Smiling Mind	Meditation	2.2 million	14	3.44	8
Relax and Sleep Well Hypnosis	Diviniti Publishing	Meditation	725 000	25	3.42	9
Headspace^c^	Headspace	Meditation	26 million	3	3.18	10

Abbreviations: ACT, acceptance and commitment therapy; CBT, cognitive behavior therapy.

^a^ A total of 46 apps with more than 1000 monthly active users and 10 000 total downloads on both marketplaces were included.

^b^ The stickiness score is the sum of the stickiness subscores for each app on the marketplaces. The stickiness subscores are the quotient of monthly active users and normalized total downloads. Normalized total download figures are the quotient of total downloads and the number of months since an app first appeared on the marketplace (it is often the case that apps are not released on both marketplaces simultaneously). Higher stickiness scores indicate stickier apps.

^c^ Indicates an app with a download rank in the top half (top 23 of 46 included apps; median [range], 1.76 [0.26-9.87]; mean [SD], 2.23 [1.84]).

## Discussion

This cross-sectional study examined download and utilization data of behavioral health apps with a focus on stickiness. We found that the most downloaded apps were not necessarily the stickiest; 7 of the 10 most downloaded were below the stickiness mean of the full included sample. We also found that some lesser known behavioral health apps (eg, Youper) are stickier than those that are widely known and downloaded (eg, Lumosity). Furthermore, our findings suggest that attributes of behavioral health apps focusing on alcohol use, smoking cessation, cognitive training, and cognitive behavioral therapy skills may be auspicious targets for future quantitative or qualitative studies on digital health engagement. While app-specific total download statistics may reflect differing levels of marketing and financial resources, we believe that the stickiness metric is more suggestive of intrinsic app-related characteristics that may be compelling to users.^4^ In a field in which few apps are used longitudinally^6^ and a panoply of engagement strategies has been tested with disappointing results,^3^ this is precisely the type of knowledge that is needed to inform app development.

Our findings are limited by the cross-sectional nature of our data; although total downloads are cumulative, the monthly active users figure is based on solely the 30 days prior to data acquisition (August-October 2018). In conclusion, future research is needed to identify key features of notably sticky apps using longitudinal methods. Such knowledge may assist in the successful real-world dissemination of evidence-based treatment models through mobile platforms and may allow the burgeoning digital health field to reach its full potential.
